# Incremental Societal Costs of Perioperative Complications Following Adult Elective Inpatient Major Therapeutic Surgery in the State of Florida: A Seven-Year Retrospective Epidemiological Analysis

**DOI:** 10.7759/cureus.62559

**Published:** 2024-06-17

**Authors:** Richard H Epstein, Franklin Dexter, Elisabeth Dexter, Brenda G Fahy

**Affiliations:** 1 Clinical Anesthesiology, University of Miami, Miami, USA; 2 Anesthesiology, University of Iowa, Iowa City, USA; 3 Surgery, Roswell Park Comprehensive Cancer Center, Buffalo, USA; 4 Anesthesiology, University of Florida, Gainesville, USA

**Keywords:** classification, health care costs, complications, medicare, diagnosis-related groups

## Abstract

Introduction

There is an expanding role for anesthesiologists in the preoperative optimization and postoperative management of patients, often in the context of a so-called perioperative surgical home. Such efforts typically include enhanced recovery after surgery (ERAS) protocols and often an anesthesiologist-led team for perioperative management. Studies of the cost-effectiveness of such approaches have generally been conducted at single institutions, with most patients cared for by small numbers of surgeons. This limitation creates generalizability issues as to whether improvement was related mostly to organizational culture or the studied surgeons' practices (non-generalizable) versus the procedures (generalizable). We studied whether other organizations can rely on achieving similar benefits following the adoption of a studied process improvement strategy at a single institution.

Methods

All patients undergoing elective major therapeutic inpatient surgery discharged between October 2015 and June 2022 at non-federal hospitals in the state of Florida were included. For each discharge, the United States Medicare Severity Diagnosis-Related Group (MS-DRG) weighting factor (i.e., the multiplier for the hospital’s base rate for admissions that determines reimbursement) and the Clinical Classification Software Refined (CCSR) code for the principal procedure were determined at admission and discharge from the state's inpatient healthcare database based on the diagnoses present at those time points. An increase in the weighting factor from admission to discharge represents societal costs from perioperative complications. Statewide, by hospital, and by surgeon, we calculated the total increase for each CCSR's weighting factor. Our primary hypothesis was that surgeon variability would be statistically greater than CCSR variability but that the incremental effect would be <5%. If CCSR and surgeon variability were comparable, this would be supportive of generalizability. In contrast, if there were a predominant effect related to the surgeon, results from one institution might not be applicable to others.

Results

Among the 1,482,344 discharges studied, the pooled (N=7 years) contributions to MS-DRG weighting factor increases from the upper 20% of surgeons were 2.8% more than from the upper 20% of CCSRs (95% CI 1.9%-3.9%, *p*=0.0006). Those CCSRs accounted for 85.5% (95% CI 79.4%-91.7%, *p*<0.0001) of the total increase in the MS-DRG weighting factor. The average contribution of the top two surgeons at each hospital to that hospital's increase in the weighting factor ranged among CCSRs from 68% to 97%. The median and 75th percentile of surgeons performing at least 10% of the total number of cases at each hospital was similar to those values for the contributions to the increases in the MS-DRG weighting factor, median 2.0 to 3.0, and 75th percentile 1.75 to 4.0.

Conclusions

Because variability among surgeons in their contributions to increases in the MS-DRG weighting factor only slightly exceeded the variability among CCSR surgical categories, perioperative surgical home and ERAS study research results involving single institutions and a small number of surgeons would likely be generalizable to other hospitals and healthcare systems. Funding agencies should not be hesitant to fund single-center perioperative surgical home studies and ERAS interventions based on concerns related to lack of generalizability.

## Introduction

There is an expanding role for anaesthesiologists to become involved in optimizing patients for surgery during the preoperative period and to participate in their postoperative care, including efforts in the United States, the United Kingdom, Canada, Australia, and New Zealand [1-3]. The concept of a Perioperative Surgical Home (PSH) is described by the American Society of Anesthesiologists (ASA) as including "improved operational efficiencies, decreased resource utilization, a reduction in length of stay and readmission, and a decrease in complications and mortality" [1]. The PSH includes the development of enhanced recovery after surgery (ERAS) protocols, such as multimodal pain therapy, prophylactic treatment of nausea and vomiting, preoperative carbohydrate loading, optimized fluid management, and avoidance of nasogastric tubes [4]. However, PSH interventions generally are more comprehensive than simply adding items to order sets in the electronic health record and providing perioperative care guidelines [5]. PSH interventions frequently include adding an anaesthesiologist-led team that evaluates patients preoperatively or follows them postoperatively during hospitalization, with cost reductions from improved patient outcomes integral to the PSH being at least net cost-neutral [5].

Efforts to validate the cost-effectiveness of such expansion of anaesthesiologists' role outside the operating room proper have generally been conducted at single institutions, with most patients cared for by small numbers of surgeons [4-20]. This observation should not be construed as a criticism but rather reflects the complexity of PSH implementation within organizations and the relatively small numbers of surgeons within each surgical subspecialty at most individual hospitals. Nonetheless, this limitation creates generalizability issues for hospitals and healthcare systems related not only to factors such as organizational culture but also to the extent to which improvement was related to studied surgeons' practices versus the procedures themselves. In other words, if a single-hospital study demonstrates the cost-effectiveness of a particular PSH or ERAS intervention, can other institutions rely on achieving similar benefits were they to implement the described process? The question is also highly relevant to funding organizations that need to decide whether to support single-center studies of such interventions or if they should rather focus on multi-center studies, where the potential confounding issues related to the study site would be mitigated.

In this retrospective, observational study of all adult patients undergoing elective, inpatient surgery who were discharged from a Florida hospital between October 2015 and June 2022, we addressed this generalizability issue by considering the incremental societal costs of perioperative complications. We determined those costs by calculating the relative increase from what would have been the United States (US) Medicare Diagnosis-Related Group (MS-DRG) weighting factor had patients not experienced a perioperative complication that increased the weighting factor. That is, using the administrative diagnosis and procedure codes for each admission, what was the fractional increase in the MS-DRG weighting factor from the expected weighting factor at the time of admission to the weighting factor calculated at discharge? We compared the variability among surgeons' total contributions for all their patients to the incremental societal cost of perioperative complications during hospitalization to the variability among each admission's principal surgical procedure, grouped by procedure categories. Our primary hypothesis was that the variability among surgeons in total increases in weighting factors would be greater, but only slightly so (<5%), than among Clinical Classification Software Refined (CCSR) procedure categories. If true, hospitals and hospital systems could reasonably infer that they would accrue value from implementing an intervention that proved beneficial at another single institution. Also, funding agencies would benefit from knowing that single-center studies of a PSH have a substantive chance of generalizability despite frequently having a few high-caseload surgeons caring for most studied patients.

## Materials and methods

The University of Miami determined (July 11, 2023) that the current analysis of de-identified publicly available data does not meet the regulatory definition of human subjects research.

Background and terminology

In this section, we briefly review terminology and hospital reimbursement in the United States because such knowledge is needed to understand our methodology. For US patients covered by Medicare (typically those 65 years and older, hospitals are reimbursed for inpatient care based on their MS-DRG. Fundamentally, that is the same process supported by the Australia Refined Diagnosis-Related Group (AR-DRG) system, through which hospital reimbursement in that country's universal healthcare coverage is provided [21,22]. Each US hospital has a blended rate, which is the average total cost of hospitalization among all admitted patients and incorporates factors such as geographic location, patient demographics, and teaching hospital status. Each MS-DRG has an associated weighting factor, which is then multiplied by the blended rate to determine the hospital reimbursement, regardless of actual costs (subject to outlier adjustment). Similarly, in Australia and New Zealand, each AR-DRG has a weight that is multiplied by the average cost of hospitalization [23]. The process incentivizes hospitals to reduce complications and decrease lengths of stay because doing so increases their margin.

All US hospitals use the same software provided by the US Centers for Medicare and Medicaid Services (CMS) to assign the MS-DRG. The MS-DRG is determined based on the primary ICD10-PCS procedure associated with the admission, comorbidities (i.e., diagnoses present at the time of admission), and complications (i.e., diagnoses that occurred during the hospitalization). MS-DRGs are typically grouped, with differences within the group related only to the presence of minor patient comorbidities and complications (CC) and major comorbidities and complications (MCC). For example, there are three codes for Craniotomy and Endovascular Intracranial Procedures: 025 (with MCC), 026 (with CC but not MCC), and 027 (without CC or MCC), with weighting factors 4.5405, 3.0235, and 2.4954. This results in higher reimbursement when patients are sicker or experience complications after hospitalization, an economically rational approach because it is more expensive to take care of sicker patients or those with complications than those who are healthy and recover uneventfully. One can infer the occurrence of hospital-acquired complications that result in increased societal costs by comparing the weighting factor corresponding to the MS-DRG that would have been assigned at the time of admission to the weighting factor corresponding to the MS-DRG assigned at the time of discharge. Because the weighting factor is multiplied by a constant (i.e., the blended rate) for each hospital, one can simply sum the changes in the weighting factors to assess the relative increase in cost due to complications.

Many patients in the US do not have Medicare, but hospitals are reimbursed for inpatient care either using MS-DRG or the similar All Patients Refined Diagnosis Related Group (APR-DRG, 3M, St. Paul, MN). The APR-DRG fundamentally follows the same reimbursement process of multiplying a weighting factor by the hospital's base rate. All hospitals in the US not administered by the government (e.g., excluding Veterans Administration hospitals) are required to report inpatients' diagnosis and procedure codes to CMS, allowing calculation of the MS‑DRG for all hospitalized patients, which defines what CMS would have paid the hospital. Thus, societal costs due to hospital-acquired complications can be estimated based on the MS-DRG weighting factor increases from admission to discharge, even though most US hospitalizations are not reimbursed by Medicare.

For grouping similar surgical admissions, we mapped the International Classification of Diseases, Tenth Revision Procedure Classification System (ICD10-PCS) code of each admission's principal surgical procedure to the corresponding CCSR code [24]. This mapping aggregates the >80,000 ICD10-PCS codes to approximately 320 categories [21].

Data sources

This dataset included publicly available data from the Agency for Health Care Administration (AHCA) obtained by the University of Florida and shared with the University of Miami under a data use agreement. As a matter of policy, AHCA disclaims responsibility for the results and conclusions of studies for which it provides data. We included records for all patients discharged from non-federal Florida hospitals between October 1, 2015 (the start of fiscal year 2016), and June 30, 2022 (the third quarter of fiscal year 2022) [25]. This date range corresponds to the first quarter where ICD-10 codes were required and the most recent available data from AHCA at the time of the study. These data were uploaded into an SQL Server database (Microsoft, Redmond, WA). All diagnosis codes were International Classification of Diseases, Tenth Revision, Clinical Modification (ICD10-CM). All procedure codes were ICD10-PCS. The Florida database provides up to 30 secondary diagnoses and 30 procedures, in addition to the primary admission diagnosis and primary procedure. Procedure classes (major or minor, and therapeutic or diagnostic) [26] and CCSR codes [4] were determined from the ICD10-PCS code listed as the principal procedure for hospitalization. Even though Medicare does not cover most patients in Florida, and there are alternative diagnosis-related group systems (e.g., the APR-DRG), Florida hospitals must submit data to the state healthcare administration, allowing the assignment of a valid MS-DRG for every hospitalized patient.

Case selection and data included

Among all 19,064,451 discharges between October 1, 2015, and June 30, 2022, we studied the 1,482,354 hospital discharges among patients ≥18 years old who underwent elective inpatient surgery with a major therapeutic procedure class (Table 1).

**Table 1:** Inclusion criteria for Florida inpatient discharges from 1 October 2015 through 6 June 2022

Group	Remaining	Excluded	Reason for exclusion
Total discharges	19,064,451		
Adults	16,870,845	(2,193,606)	<18 years old
Adults having major therapeutic surgery	3,911,095	(12,959,750)	Primary surgery was not major therapeutic surgery
Adults having major therapeutic surgery on the day of admission	2,236,590	(1,674,505)	Primary surgery was not on the day of admission
Adults having major therapeutic surgery on the day of admission, not urgent or emergent	1,495,393	(741,197)	Surgery was urgent or emergent
Adults having major therapeutic surgery on the day of admission, not urgent or emergent, not admitted via the emergency department	1,482,354	(13,039)	The patient was admitted through the emergency department
Total analyzed	1,482,354		

We limited our analysis to adults because the MS-DRG categories are known not to apply well to children [27], to inpatients because MS-DRG codes do not apply to outpatients, to elective cases because urgent and emergent surgery do not present potential opportunities for preoperative optimization in a PSH, and to major therapeutic surgery because those cases uniformly involve anesthesia services [28-30]. Patients undergoing care at federal hospitals (e.g., Veterans Administration hospitals) do not report their data to the state, so they could not be included. The data fields required by the MS-DRG Grouper Software (see previous section) were extracted into a text file, with each admission in a separate row having the required 835-character fixed-format string. Secondary diagnoses 25 to 30 in the AHCA dataset were ignored if present.

MS-DRG Grouper Software implementation details

This section is provided for readers wishing to fully understand or replicate our methodology of calculating changes in the MS-DRG weighting factor using the primary procedure and diagnosis information present at the time of admission and the additional diagnoses (some of which represent complications) from the entire hospitalization. The section can be skipped for those without such interests.

The MS-DRG Grouper Software and Medicare Code Editor Version 40, ICD-10 PC Software (MS Grouper software) was obtained from the CMS [31]. This software, compatible with Windows® 8.1 or later (Microsoft, Redmond, WA), comprises an interactive and batch version written in Java (Oracle, Redwood Shores, CA) and applies the relevant MS-DRG grouper versions and ICD codes for the fiscal year corresponding to the discharge date. This grouper software, used for US Medicare billing, requires a primary diagnosis and up to a maximum of 24 secondary diagnoses, with optional annotation as to whether the diagnosis was present on admission, one primary and up to 24 secondary procedures, the date of discharge, discharge status (e.g., home or self-care), and the patient's age and sex. Fields such as sex and age are not used directly to assign the MS-DRG but rather to check that procedures and diagnoses listed are consistent with those elements (e.g., an error code would be returned if a male patient was listed as having a hysterectomy or if a female was listed as having benign prostatic hypertrophy). The discharge date is used to apply the logic and MS-DRG weighting factor for each fiscal year's Medicare codes. For MS-DRG assignment purposes, only the principal procedure is used, with the other procedure codes used to check for inconsistencies with other data elements. The batch version of the MS-DRG grouper software was used for analysis, and the interactive version was used to check a convenience sample of results.

Each record from the Florida database was represented by two rows, the first with all diagnoses and procedures listed for the hospitalization and the second with only the diagnoses present on admission and the procedures performed on the day of admission. From these data, the final MS-DRG was calculated using the MS Grouper Software. These data were exported to a text file, and the data fields were parsed by software written in Python version 3.10.5 (under the Integrated Development and Learning Environment (IDLE, Python Software Foundation, Beaverton, OR)). In addition, for hospitalizations where the final MS-DRG from the Grouper Software based on all data was different from the final MS-DRG similarly calculated using only the present-on-admission information, a new file was created using the present-on-admission data as the base information and then adding each diagnosis that was not present on admission one at a time. Thus, each row included the present-on-admission ICD10-CM codes and one additional ICD10-CM code for a hospital-acquired condition, some of which represented complications. This approach allowed a determination of whether the added diagnosis was responsible for the escalation of the MS-DRG from admission to discharge and, thus, for the corresponding increase in the MS-DRG weighting factor. The ICD10-CM codes responsible for escalation were considered to represent a complication during hospitalization, changing the MS-DRG from one without complications or comorbidities (without CC/MCC) to one with major comorbidities or complications (with CC or with MCC), or from one with complications (with CC) to one with major comorbidities or complications (with MCC).

Validation of the MS-DRG determination using our implementation of the Grouper Software

To validate our implementation of the MS-DRG grouper software, we compared the MS-DRG in the Florida database to the calculated MS-DRG among 1,482,344 adult patients discharged following inpatient elective surgery over the six-year, nine-month interval between October 2015 and June 2022. There was 99.97% concordance between the MS-DRG listed in the Florida database and the final DRG reported by the MS Grouper Software using the diagnosis and procedure information in the database. Thus, by inference, our use of the grouper software to determine the MS-DRG using the information known at the time of admission was validated.

Calculation of the incremental cost in weighted units due to hospital complications

We estimated the total societal cost of complications among all inpatients by determining the total increase in the MS-DRG weighting factors resulting from perioperative complications, where N=1,482,354 studied discharges and the weighting factors were those calculated at admission and discharge (Equation 1).

$$Total\: Societal\: Cost = \sum_{i=1}^{N}\left ( [Weighting\: Factor\: at\: Discharge]_{i} - [Weighting\: Factor\: at\: Admission]_{i} \right )\: \; \; \; \; \; \; \; \; \; \; (1)$$

The change in the US MS-DRG weighting factor is a valid estimate of increased societal cost based on the US Medicare prospective payment system, for many decades, calculating hospital reimbursement as the product of the hospital's blended rate per case (a fixed amount) and the weighting factor corresponding to the MS-DRG [32]. The DRG reimbursement model has also been applied by non-Medicare payors to nearly all adult acute care hospitals in the USA. The blended rate calculated for each hospital is influenced by various factors unrelated to the individual patient, including location, local population demographics, and teaching hospital status. The higher weighting factor for patients with comorbidities or complications reflects the increased cost of care for such patients compared to healthy patients with no complications. Because we are dealing with increases relative to the fixed blended rate for each hospital, differences in the MS-DRG weighting factor among different procedures are incorporated. For example, at a given hospital, an increase in the weighting factor for a relatively low-reimbursement MS‑DRG from 0.5 to 1.0 is the same as an increase from a weighting factor of 3.0 to 3.5 for a high‑reimbursement MS-DRG.

For example, consider an otherwise healthy 75-year-old patient with prostate cancer admitted for an open radical prostatectomy (ICD10-PCS 0VT00ZZ) in 2018. From the information on the day of surgery, the MS-DRG code would be 708 (major male pelvic procedures without CC/MCC), and the MS-DRG weighting factor is 1.4065. If he had an uneventful postoperative course, that would also be the discharge MS-DRG. However, suppose he developed acute renal failure with acute tubular necrosis (N170) during the hospitalization. Then, the MS-DRG would be changed to 707 (major male pelvic procedures with CC/MCC) with an MS-DRG weighting factor of 1.7914. The incremental increase in weighted units resulting from this complication would be 0.3849 (=1.7914-1.4065). If the hospital's blended rate were $6000 per case, the estimated societal cost of the complication would be $2309 (=0.3849x$6000).

For each admission, we determined the incremental societal cost in weighted units attributable to conditions acquired during hospitalization based on the change in the MS-DRG that would have applied using only the principal procedure and comorbidities present on admission to the final MS-DRG using all the diagnoses and the principal procedure. If there was no MS-DRG change or if the change did not alter the MS-DRG weighting factor, the incremental societal cost was treated as 0. Most of the MS-DRG surgical codes are grouped for patients having similar procedures, distinguished by the presence or absence of CC or the presence of MCC. For most groups of related MS-DRG codes, the lowest level code is for a patient without CC. For example, consider a 75-year-old patient with chronic systolic (congestive) heart failure (ICD10 I5022) undergoing an open radical prostatectomy for prostate cancer. At admission, he would already be assigned MS-DRG 707 (with MCC) because congestive heart failure is a major comorbidity. The expected occurrence rate of complications is built into the MS-DRG weight. If that patient develops postoperative renal failure, the MS-DRG would remain the same. In this scenario, there would be no increase in the estimated incremental societal cost of the complication because the total cost of caring for such patients with pre-existing major comorbidities was already incorporated into the weighting factor for the MS-DRG. Thus, if the patient were reimbursed under traditional Medicare, there would be no added CMS reimbursement to the hospital. From this, one should not infer that hospitals forego attempts to reduce complications in patients with major comorbidities present on admission. Similar processes are in place for activity-based reimbursement based on other diagnosis-related group methodologies in the United States (e.g., the APR-DRG system) and in other countries such as Australia, which uses the AR-DRG system to classify hospitalizations.

Validation of our implementation of the MS-DRG Grouper Software

To validate our implementation of the MS-DRG Grouper software, we compared the final DRG reported by that software to the MS-DRG recorded in the Florida database using all relevant data from the hospitalization (i.e., all procedures and diagnoses). Because the MS-DRGs in the Florida database are determined by the Grouper Software from the same diagnoses, principal procedure, and demographic information provided by the hospitals in the database, we expected close to 100% concordance. A small error rate was expected due to transcription errors and potentially from excluding diagnoses noted beyond the first 24 listed in the Florida database.

Comparison of inequalities of contribution to the escalation in the MS-DRG by CCSRs compared to surgeons (primary hypothesis)

We compared the inequalities in the contribution of surgeons and CCSR codes to the total fractional increase in the MS-DRG weighting factor. For each combination of the seven fiscal years, percentile shares were calculated using the Stata function pshare to estimate the mean and variance of the total fractional increase attributable to the 20% of the surgeons and to the CCSR codes accounting for the largest fractional increases. For each of the N=7 years, the differences between these fractional increases and their standard errors were calculated from the point estimates and the corresponding variances. The overall estimate of the difference (surgeons minus CCSR codes) among the fiscal years was calculated using a DerSimonian-Laird random effects model with Knapp-Hartung adjustment (Stata *meta *command). A two-sided P-value and the 95% confidence of the pooled difference between the groups were calculated.

Determination of the numbers of surgeons at hospitals contributing to increases in the MS-DRG weighting factor in fiscal year 2021 (secondary analysis #1)

We studied the fiscal year 2021 (October 1, 2020, through June 30, 2021) because this was both representative and the most recent year for which all four quarters of data were available from AHCA. We first determined the top 10 CCSRs responsible for the total increase in the MS-DRG weighting factor, statewide, where N_CCSR_ refers to the number of discharges for each of the specified CCSRs (Equation 2).

$$CCSR\: \: Cost = \sum_{i=1}^{N_{CCSR}}\left ( [Weighting\: Factor\: at\: Discharge]_{i} - [Weighting\: Factor\: at\: Admission]_{i} \right )_{CCSR}\: \; \; \; \; \; \; \; \; \; \; (2)$$

We then determined, for each of these CCSRs, the hospitals in Florida that cumulatively contributed 80% to the total increase. For each of these CCSRs and included hospitals, we calculated the relative contribution to the increase in the MS-DRG weighting factor by each surgeon performing procedures in the CCSR, where Surgeon⋅CCSR refers to the combination of surgeon and CCSR and N_Surgeon⋅CCSR_* *refers to the number of discharges for the specified combination (Equation 3).

$$Surgeon\cdot CCSR\: \: Cost = \sum_{i=1}^{N_{Surgeon\cdot CCSR}}\left ( [Weighting\: Factor\: at\: Discharge]_{i} - [Weighting\: Factor\: at\: Admission]_{i} \right )_{Surgeon\cdot CCSR}\: \; \; \; \; \; \; \; \; \; \; (3)$$

Next, for each CCSR and included hospital, we determined (i) the number of surgeons who contributed at least 10% to the hospital's total increase in MS-DRG weighting factors and (ii) the percentage of the total increase attributable to the two highest contributing surgeons, where Hospital.⋅CCSR refers to the combination of Hospital and CCSR and N_Hospital.⋅CCSR_ refers to the number of discharges for the specified combination (Equation 4).

$$Hospital\cdot CCSR\: \: Cost = \sum_{i=1}^{N_{Hospital\cdot CCSR}}\left ( [Weighting\:Factor\: at\: Discharge]_{i} - [Weighting\: Factor\: at\: Admission]_{i} \right )_{Hospital\cdot CCSR}\: \; \; \; \; \; \; \; \; \; \; (4)$$

Then, among all included hospitals for each of the 10 CCSRs, we calculated (i) the median and interquartile range for their numbers of surgeons and (ii) the mean and standard deviation of the percentage contributions to the increases in the MS-DRG weighting factor from the top two surgeons.

Calculation of percentile shares for CCSRs and the causative diagnoses among cases with MS-DRG escalation (secondary analysis #2)

To address the generalizability issue, we first needed to confirm that our approach of using complication-associated increases in the MS-DRG weighting factor identified classes of surgical procedures that have been frequent targets of interventions to reduce perioperative complications (i.e., to validate that our method has face validity). We identified the categories of inpatient elective surgical procedures responsible for 80% of the total cost increase attributable to hospital-acquired conditions. Our second hypothesis was that fewer than 20% of the CCSR surgical categories would account for at least 80% of the added costs. The total added cost among all inpatients was inferred from the increases in the MS-DRG weighting factor applied comparing (i) only the comorbidities present on admission and the principal procedure performed to (ii) the discharge MS-DRG using all diagnoses and the principal procedure from the hospitalization. Such knowledge of the CCSR accounting for most of the total incremental costs is important for researchers because it will help focus their studies on the procedures and interventions that can substantively improve overall population-based patient care. Analysts can apply the methodology we describe to help administrators at individual healthcare systems optimally deploy resources (e.g., hospitalists, PSH personnel) to decrease their hospitals' healthcare costs and lengths of stay.

We identified each record where the MS-DRG calculated by the grouper using all diagnoses and procedures differed from the MS-DRG calculated using only diagnoses noted as present on admission and the principal procedure. To determine which CCSRs were primarily responsible for these MS-DRG escalations among all patients, we grouped these records by CCSR and determined the total increase in the MS-DRG weighting factor due to the change in the MS-DRG. In other words, all the increases in MS‑DRG weighting factors were summed among all discharges with the CCSR for the principal procedure. Among the 327 CCSRs, we included the 189 categories for which at least one major therapeutic procedure was listed as the principal procedure for any discharge in the CCSR among all cases in the entire dataset. For each CCSR, we also computed a caseload adjustment factor, which was the quotient of the total number of hospitalizations for that CCSR divided by the total number of hospitalizations among all the CCSRs (i.e., the total number of discharges)), where N represents the total number of discharges and N_CCSR_ the number of discharges with the specified CCSR (Equation 5).

$$Caseload\: Adjustment\: Factor_{CCSR}= \frac{N_{CCSR}}{N}\; \; \; \; \; \; \; \; \; \; (5)$$

The percentile share for each CCSR was analyzed using the pshare command in Stata v17.0 (Stata Corp, College Station, TX) [33]. Standard errors of the percentile shares were calculated using the bias-corrected and accelerated bootstrap with 1000 replications. This process was performed with and without the caseload adjustment factor. Importantly, percentile shares are analyzing, as appropriate, the totals among all discharges for each CCSR, thus incorporating variability both in the mean increases in weights for each CCSR and the variability in the numbers of discharges among CCSRs. We did not perform an analysis such as generalized linear modeling followed by estimation of the intra-cluster correlation because that would essentially analyze the mean increase in the MS-DRG weighting factor for each CCSR and then the variability among means, neglecting the inequality in the number of discharges among the CCSRs. Similarly, we did not use the mean increase in the MS-DRG weighting factor for each CCSR and its standard error to estimate an overall weighted increase because inequality in the numbers of discharges among CCSRs is central to the hypothesis.

Contributions of ICD10-CM diagnoses to the incremental societal cost of complications (secondary analysis #3)

From the list of ICD10-CM diagnoses responsible for the escalation in the MS-DRG from admission to discharge, we determined the contribution of each diagnosis to the percentage of cases where the MS-DRG changed. Note that this secondary analysis estimates incremental costs and will systematically underestimate the incidence of postoperative complications. For example, if the patient were already in an MS-DRG with MCC and developed acute renal failure, a major complication, that would not be recorded as an incremental societal cost because the MS-DRG, and thus hospital reimbursement, would not have changed.

Statistics

Statistical tests were performed with Stata version 17.0. P‑values<0.05 were required for statistical significance, and 95% confidence intervals were reported. Distributions of variables were summarized using median and interquartile ranges, using the *percentile.exc* function in Excel (Microsoft), which corresponds to the R-6 method described by Hyndman and Fan [34,35]. Because every adult inpatient having elective surgery in a Florida hospital and discharged during the study interval was included, the dataset does not represent a sample; thus, an a priori power analysis was not performed.

## Results

Relative contributions of CCSRs versus surgeons to MS-DRG weighting factor increases (primary hypothesis)

**Figure 1:**
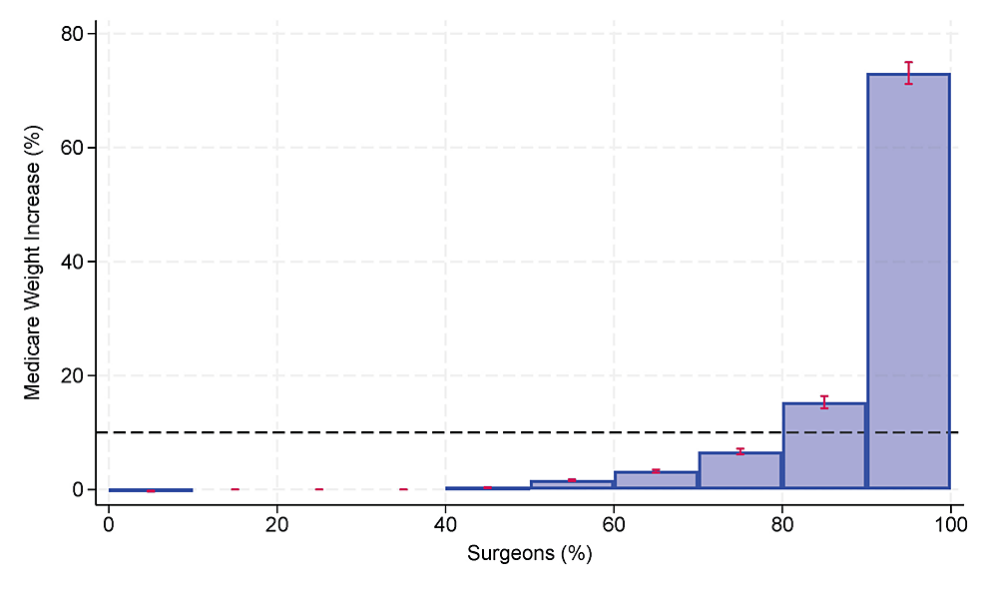
Proportional shares among deciles of surgeons for the percentage increase in MS-DRG weighting factors due to complications in adult patients undergoing elective major therapeutic inpatient surgery in Florida during 2019, a typical year. The top decile, representing the 10% of surgeons with the greatest contribution, accounted for 73.1% (95% CI 71.2% to 75.0%) of the total increase. The dashed line at 10% represents the line of equality if all quintiles had the same contribution. The histogram shows substantive inequality among the surgeons. MS-DRG: Medicare Severity Diagnosis-Related Group

For each of the seven fiscal years (2016 to 2022), there was substantive inequality among surgeons in their contribution to the total increases in the MS-DRG weighting factor. For example, in 2019, a representative year, the upper 10% of surgeons were responsible for 73.1% (95% CI 71.2% to 75.0%) of the total increase (Figure 1), while the upper 20% of surgeons were responsible for 88.4% (95% CI 87.5% to 89.3%). To put this inequality into perspective, we compared the contributions of the upper 20% of surgeons to that of the upper 20% of CCSRs. The pooled difference in the contributions to the increase in the MS-DRG weighting factors over the seven-year study interval from the upper 20% of surgeons compared to the upper 20% of CCSRs was 2.8% (95% CI 1.9% to 3.9%, p=0.0006). The heterogeneity coefficient I2 for the weighted differences among the N=7 years was 0.0%, justifying pooling the results. As we hypothesized, there was a slightly greater inequality, but less than 5%, contributed by differences among surgeons versus differences among CCSRs.

Number of surgeons at hospitals contributing to increases in the MS-DRG weighting factor in fiscal year 2021 (secondary analysis #1)

For the top 10 CCSRs in fiscal year 2021 that contributed statewide to increases in the Medicare weighing factor, among the hospitals that contributed 80% to the statewide increase for each CCSR, the median number of surgeons contributing to at least 10% of the total increase for the CCSR and hospital ranged from 2.0 to 3.0 surgeons and the 75th percentile from 2.0 to 4.0 surgeons. The average contribution of the top two surgeons at each hospital to that hospital's increase in the MS-DRG weighting factor ranged among CCSRs from 68% to 97% (Table 2). The median and 75th percentile of surgeons performing at least 10% of the total number of cases at each hospital was similar to those values for the contributions to the increases in the MS-DRG weighting factor, median 2.0 to 3.0 and 75th percentile 1.75 to 4.0. These results confirm the conditions motivating the testing of the primary hypothesis that single hospital studies of the PSH will be studies of a few surgeons.

**Table 2:** Example of the epidemiology of surgery among surgical procedure categories in the state of Florida in 2021 CCSR: Clinical Classifications Software Refined surgical procedure code; MS-DRG: Medicare Severity Diagnosis-Related Group 1. The CCSRs included were the top 10 contributing to the total increase in the MS-DRG weighting factor in Florida during Fiscal Year 2021 among adults undergoing elective, inpatient major therapeutic surgery where there was a change in the MS-DRG calculated using information available at the time of admission to the MS-DRG using all diagnosis and procedure information for the hospitalization. That is, these are the most important CCSRs with perioperative complications. 2. Percentage contribution among all facilities in the state for the listed CCSR of the increase in the MS-DRG weighting factor to the total increase among all CCSRs. The CCSRs are listed in decreasing order of their contributions. 3. Within each hospital that contributed to 80% of the total increase in the MS-DRG weighting factor for the listed CCSR, the number of surgeons at that facility who contributed at least 10% to that hospital's contribution to the increase in the MS-DRG weighting factor was determined. Then, the median and IQR among all these hospitals were determined. 4. Within each hospital that contributed 80% of the total increase in the MS-DRG weighting factor for the listed CCSR, the percentage contribution of the top two surgeons to that hospital's contribution was determined. Then, the mean and standard deviation were calculated among all these hospitals. 5. Within each hospital that contributed to 80% of the total increase in the MS-DRG weighting factor for the listed CCSR, the number of surgeons at that facility who performed at least 10% of the cases was determined. Then, the mean and standard deviation were calculated among all these hospitals.

CCSR^1^	Description	% Contribution to the total increase in the MS-DRG weighting factor, statewide^2^	# Surgeons median (IQR)^3^	Contribution to the increase in the MS-DRG weighting factor of the top two surgeons within each facility mean (SD) # facilities^4^	# Surgeons performing at least 10% of the cases median (IQR)^5^
MST013	Spine fusion	14.7%	3 (2,4)	75% (SD 16%) N=53	3 (2,4)
CAR022	Heart valve replacement and other valve procedures (non-endovascular)	13.9%	2 (1,3)	85% (SD 13%) N=22	2 (2,3)
GIS009	Colectomy	7.0%	3 (3,4)	72% (SD 18%) N=79	3 (2,4)
CAR003	Coronary artery bypass grafts (CABG)	6.3%	2 (2,3)	85% (SD 16%) N=32	3 (2,3)
RES008	Lung, pleura, or diaphragm resection (open and thoracoscopic)	5.7%	2 (1,2)	93% (SD 10%) N=18	2 (2,3)
MST006	Knee arthroplasty	3.0%	3 (2,4)	77% (SD 19%) N=49	3 (2,4)
MST007	Hip arthroplasty	2.0%	2 (2,3)	84% (SD 18%) N=49	3 (2,4)
FRS001	Hysterectomy	2.0%	3 (2,4)	66% (SD 20%) N=44	3 (2,4)
CAR006	Carotid endarterectomy and stenting	2.0%	2 (2,3)	82% (SD 21%) N=41	3 (2,4)
CAR012	Vessel repair and replacement	2.0%	2 (1,3)	86% (SD 17%) N=16	2 (2,4)

Percentile shares for CCSRs and the causative diagnoses among cases with MS-DRG escalation (secondary analysis #2)

There was stability (i.e., no trend over time) in the top 10 CCSRs associated with changes in the MS‑DRG calculated (i.e., increase in the MS-DRG weighting factor) using only the admission data versus all diagnosis and procedure data for the hospitalization between fiscal years 2016 and 2022. There also was no trend among the seven fiscal years in the total increase in the weighting factor of the top 10 CCSRs, 57.9% (SD 0.8%). Thus, we pooled the CCSR data to determine the surgical categories mostly responsible for the increases in the MS-DRG weighting factor (Table 3). The most common CCSRs, which cumulatively accounted for just over half of the increase in the MS-DRG weighting factor, in descending order of the point estimates, were spine fusion, heart valve surgery, colectomy, pulmonary resection, coronary artery bypass graft, and hip and knee arthroplasty (Table 3). These CCSRs include the surgical procedures commonly targeted for interventions to reduce complications [2-19], confirming the face validity of our method of using the MS‑DRG weighting factor to measure incremental societal costs of complications. The impact of each additional case with an escalation in the MS-DRG differed greatly among the CCSRs. For example, the overall contribution from spine fusion and invasive heart valve surgery was similar, 13.1% and 12.7%, respectively, even though six times as many fusions were performed.

**Table 3:** Percent contribution by the CCSRs responsible for 80% of the total increase in the MS-DRG weighting factor CCSRs: Clinical Classification Software Refined; MS-DRG: Medicare Severity Diagnosis-Related Group

CCSR	Description	Clinical domain	#Discharges	Contribution	Cumulative contribution
MST013	Spine fusion	Musculoskeletal, Subcutaneous Tissue, and Fascia Procedures	133,607	13.1%	13.1%
CAR022	Heart valve replacement and other valve procedures (non-endovascular)	Cardiovascular Procedures	22,216	12.7%	25.8%
GIS009	Colectomy	Gastrointestinal System Procedures	60,633	7.6%	33.4%
RES008	Lung, pleura, or diaphragm resection (open and thoracoscopic)	Respiratory System Procedures	25,546	5.6%	39.0%
CAR003	Coronary artery bypass grafts (CABG)	Cardiovascular Procedures	20,009	5.6%	44.6%
MST006	Knee arthroplasty	Musculoskeletal, Subcutaneous Tissue, and Fascia Procedures	211,433	4.0%	48.6%
MST007	Hip arthroplasty	Musculoskeletal, Subcutaneous Tissue, and Fascia Procedures	148,261	2.6%	51.2%
FRS001	Hysterectomy	Female Reproductive System Procedures	47,982	2.2%	53.4%
CAR006	Carotid endarterectomy and stenting	Cardiovascular Procedures	36,284	2.0%	55.5%
URN008	Nephrectomy and ureterectomy	Urinary System Procedures	23,822	1.8%	57.3%
GIS010	Gastrectomy	Gastrointestinal System Procedures	54,952	1.8%	59.1%
GNR002	Abdominal wall repair (including hernia)	General Region Procedures	16,060	1.8%	60.8%
CAR012	Vessel repair and replacement	Cardiovascular Procedures	3,459	1.7%	62.5%
PGN003	Cesarean section	Pregnancy-Related Procedures	192,155	1.7%	64.2%
CAR023	Heart valve replacement and other valve procedures (endovascular)	Cardiovascular Procedures	26,261	1.6%	65.7%
CAR011	Aneurysm repair procedures	Cardiovascular Procedures	17,354	1.5%	67.2%
HEP005	Pancreatectomy	Hepatobiliary and Pancreas Procedures	6,195	1.5%	68.7%
RES011	Diaphragmatic hernia repair	Respiratory System Procedures	7,590	1.4%	70.1%
GIS011	Small bowel resection	Gastrointestinal System Procedures	11,407	1.3%	71.4%
URN009	Cystectomy (including fulguration) and urethrectomy	Urinary System Procedures	6,328	1.2%	72.6%
CAR014	Peripheral arterial bypass procedures	Cardiovascular Procedures	10,147	1.2%	73.8%
GIS026	Upper GI therapeutic procedures, NEC (open and laparoscopic)	Gastrointestinal System Procedures	7,936	1.1%	74.9%
CNS007	CNS excision procedures	Central Nervous System Procedures	9,462	0.9%	75.8%
GIS014	Esophagectomy	Gastrointestinal System Procedures	1,423	0.9%	76.7%
HEP004	Hepatobiliary resection and ablation	Hepatobiliary and Pancreas Procedures	4,516	0.8%	77.5%
GIS013	Proctectomy or anal resection	Gastrointestinal System Procedures	5,618	0.7%	78.3%
GIS022	GI system lysis of adhesions	Gastrointestinal System Procedures	4,940	0.7%	79.0%
GIS019	Gastro-jejunal bypass (including bariatric)	Gastrointestinal System Procedures	18,746	0.7%	79.7%
CAR019	Septal repair and other therapeutic heart procedures	Cardiovascular Procedures	2,526	0.7%	80.3%

**Figure 2:**
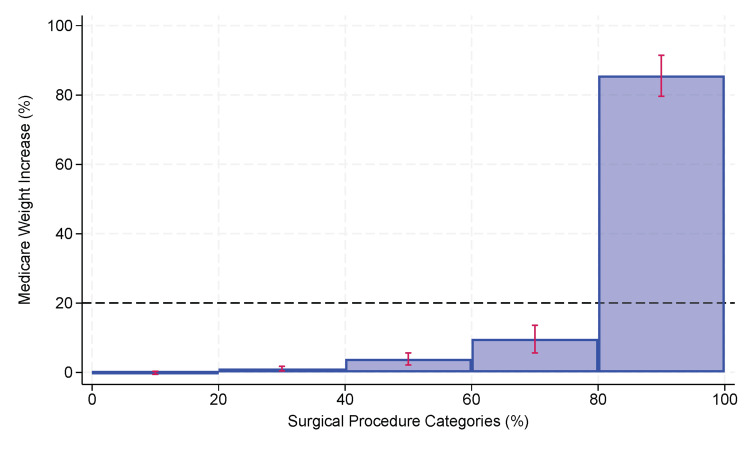
Proportional shares among quintiles of surgical procedure categories for the percentage increase in MS-DRG weights due to complications in adult patients undergoing elective major therapeutic inpatient surgery in the state of Florida between October 1, 2015, and June 30, 2022. Procedure category CCSR codes were determined from the ICD10-PCS code of the principal procedure during the hospitalization. The top quintile, representing the 20% of CCSRs with the greatest contribution, accounted for 85.5% (95% CI 79.4% to 91.7%) of the total increase. The dashed line at 20% represents the line of equality if all quintiles had the same contribution. The histogram shows substantive inequality among the CCSRs. CCSR: Clinical Classification Software Refined; ICD10-PCS: International Classification of Diseases, Tenth Revision, Procedure Classification System; MS-DRG: Medicare Severity Diagnosis-Related Group

The inequality among all CCSRs in their contributions to the total increase in MS-DRG weighting factors is shown graphically in Figure 2, where 20% of the top CCSRs were responsible for 85.5% (95% CI 79.4% to 91.7%, p<0.0001 compared to 20%) for the total increase in MS-DRG weighting factors in the entire state. As we hypothesized, fewer than 20% of the CCSR surgical categories accounted for at least 80% of the added societal costs.

Contributions of ICD10-CM diagnoses to the incremental societal cost of complications (secondary analysis #3)

Table 4 shows the diagnoses responsible for the escalation of the MS-DRG from admission to discharge. The complications occurring in at least 1% of cases represent a diverse group of organ systems. Among the 3729 ICD10-CM hospital-acquired conditions causing an escalation in the MS-DRG, the most frequent 84 (2.3% 95% CI 1.8% to 2.7%) were responsible for 80% of the occurrences.

**Table 4:** ICD10-CM codes most frequently resulting in MS-DRG escalation MS-DRG: United States Medicare Severity Diagnosis-Related Group; ICD10-CM: International Classification of Diseases, Tenth Revision, Clinical Modification

ICD10-CM	Description	Organ system	#Discharges	Contribution	Cumulative contribution
D62	Acute posthemorrhagic anemia	Hematologic	38795	20.3%	20.3%
J9601	Acute respiratory failure with hypoxia	Pulmonary	7911	4.1%	24.4%
K567	Ileus, unspecified	Gastrointestinal	7817	4.1%	28.5%
J9811	Atelectasis	Pulmonary	5608	2.9%	31.5%
N179	Acute renal failure, unspecified	Renal	5378	2.8%	34.3%
J95821	Acute postprocedural respiratory failure	Pulmonary	4741	2.5%	36.8%
E871	Hypo-osmolality and hyponatremia	Renal	4334	2.3%	39.0%
J189	Pneumonia, unspecified organism	Pulmonary	4199	2.2%	41.2%
N170	Acute kidney failure with tubular necrosis	Renal	3530	1.8%	43.1%
J951	Acute pulmonary insufficiency following thoracic surgery	Pulmonary	3225	1.7%	44.8%
A419	Sepsis, unspecified organism	Sepsis	2878	1.5%	46.3%
J690	Pneumonitis due to inhalation of food and vomit	Pulmonary	2666	1.4%	47.7%
G92	Toxic encephalopathy.	Neurologic	2551	1.3%	49.0%
G9341	Metabolic encephalopathy	Neurologic	2096	1.1%	50.1%
K9189	Other postprocedural complications and disorders of digestive system	Gastrointestinal	2027	1.1%	51.2%
O721	Other immediate postpartum haemorrhage	Hematologic	1945	1.0%	52.2%
J9600	Acute respiratory failure, unspecified whether with hypoxia or hypercapnia	Pulmonary	1908	1.0%	53.2%
R571	Hypovolemic shock	Cardiac	1880	1.0%	54.1%
J9602	Acute respiratory failure with hypercapnia	Pulmonary	1647	0.9%	55.0%
E872	Acidosis	Sepsis	1573	0.8%	55.8%
J952	Other and unspecified premature depolarization	Cardiac	1494	0.8%	56.6%
J90	Pleural effusion, not elsewhere classified	Pulmonary	1455	0.8%	57.4%

## Discussion

Most studies of the PSH and ERAS protocols have involved one or only a few hospitals, which is likely to continue [4-19]. The consequence is that, in practice, interventions often have been applied principally to a few surgeons' patients. Our data from Table 2 demonstrate that such concentration is an inescapable consequence of the epidemiology of subspecialty surgical care: relatively few surgeons at any hospital contribute substantively to the hospital's increase in the MS-DRG weighting factor due to perioperative complications, and relatively few surgeons frequently perform procedures within a given CCSR. Regardless of whether the title of a research study states that joint arthroplasty patients have been studied and the abstract refers to joint arthroplasty, if the hospital studied has three surgeons who performed 80% of those cases, what has been studied are three surgeons' patients. If improvements in patient care from PSH interventions were to be mostly driven by changes in individual surgeons' practices rather than related to the studied procedures, such studies might have limited generalizability to other hospitals and healthcare systems.

Recent studies have highlighted the large impact of unmeasured covariates from practice differences among surgeons [36]. Statistical adjustments can be made to compensate within studies for such heterogeneity of practices. However, that does not address the broader issue of the generalizability and strategic value of research in the PSH, which we addressed in the current study. Because surgeons are nested within categories of procedures, there inevitably will be greater inequality among surgeons in the extra societal costs accruing from perioperative complications than among categories of procedures. Our sample size of seven years of data from the state of Florida was sufficient to reliably detect this difference between the contributions of surgeons and procedures, p=0.0006. Our important result was learning that the magnitude of the difference between surgeons and procedure categories was small, approximately 2.8%, 88.7% versus 85.8%, satisfying our primary hypothesis.

From a policy and research perspective related to studies of the PSH, which typically involves patients undergoing procedures in multiple CCSRs, attention should be on the 85.8% of extra healthcare resources attributable to 20% of inpatient surgical procedures, our first secondary analysis. That is an important consideration for organizations funding such research, such as the Anesthesia Patient Safety Foundation [37], the Foundation for Anesthesia Education and Research [38], and the Health Services Research Association of Australia and New Zealand [39].

The finding that multiple organ systems were implicated in complications that increase societal costs makes it unlikely that the sole involvement of a specialist (e.g., pulmonologist, cardiologist) would have a substantive impact on the overall reduction [40]. Rather, the involvement of a physician hospitalist-directed team to coordinate postoperative medical care would be a more rational approach. For patients admitted to an intensive care unit (ICU), the involvement of a critical care physician during that interval of care would also be reasonable. However, most postoperative inpatients are not admitted to an ICU, and ICU lengths of stay are typically short.

Complications during hospitalization among adult patients having elective major therapeutic surgery occur disproportionately in a few procedure categories, as defined by the CCSR coding system. For example, the total increase in healthcare resources from heart valve replacement and spinal fusion accounts for more than 10%, whereas the contribution from craniotomy for tumor excision was less than 1% (Table 3). The most common CCSRs we identified where complications resulted in MS-DRG escalation are conceptually logical and unsurprising. However, the quantitative information we provide can help researchers ensure that they are studying population-important procedures and provide the data needed for grant proposals to study individual procedures.

Limitations and strengths

This study included data from only a single state in the United States. However, Florida is highly populated and has a diverse population with varying access to healthcare. Furthermore, we studied every elective inpatient major therapeutic procedure in adults over a seven-year interval. Because MS-DRGs are not assigned for outpatient procedures, we cannot generalize our findings to outpatient procedures even though they may be included in the same CCSR as an inpatient procedure. For example, hip and knee arthroplasties are increasingly being performed on an outpatient basis (i.e., less than two-day length of stay), but such patients are selected based on a risk assessment score [8,41]. Our results should not be applied to pediatric patients who were not studied and to whom the MS-DRG weighting system is not applicable [24]. Furthermore, results may not be generalizable to patients who receive care in federal hospitals (e.g., Veterans Administration hospitals) because they were not represented in the dataset, and such patients' complications may differ from patients in other hospitals. We could only apply calculations based on the MS Grouper Software, not on the APR-DRG algorithms, because 3M does not publicly disclose its methodology. Furthermore, the APR-DRG incorporates disease severity-related factors unavailable in the state dataset. However, the APR-DRG system is fundamentally the same as the MS-DRG system, incorporating a weighting factor affected by hospital complications that is multiplied by the hospital's base rate to determine reimbursement. Therefore, it is reasonable to expect that the calculation of societal costs based on changes in the APR-DRG between admission and discharge would produce results qualitatively similar to our study. A strength of the study is that Florida hospitals are required by state law to provide the complete data required for calculating the MS-DRG to AHCA for every patient admitted to their facility. Finally, DRG-based reimbursement systems are similar worldwide, based on average costs with adjustments for patients with relevant comorbidities and complications that increase the cost of care. Thus, we would expect that the findings would be applicable to Canada, the United Kingdom, Australia, and New Zealand.

## Conclusions

In adults undergoing inpatient elective major therapeutic surgery, more than 85% of the increases in societal healthcare costs attributable to perioperative complications that resulted in an increase in the MS-DRG weighting factor were attributed to only 20% of the surgical procedure categories. Most of this inequality was related to differences among the CCSR procedure codes rather than from the surgeons performing the procedures. The principal implication is that studies of the PSH for groups of surgical procedure categories involving single or small numbers of hospitals and relatively few surgeons are likely to be generalizable to other healthcare systems. Because the hospital-acquired conditions responsible for 80% of the cases in which complications caused an increase in the MS-DRG weighting factor were distributed among many organ systems, strategies to mitigate their occurrence likely should primarily involve hospitalists rather than medical subspecialists. These factors should help focus attention by hospital administrators, researchers, and funding agencies to support efforts with the greatest potential to reduce societal healthcare costs.
